# THE PRESCRIPTION DRUG LANDSCAPE OF ALZHEIMER’S DISEASE AND RELATED DEMENTIAS

**DOI:** 10.1093/geroni/igac059.1817

**Published:** 2022-12-20

**Authors:** Lauren Bangerter, Callahan Clark, Megan Jarvis, Emma Coates, Natalie Sheils, Alyssa Wong, Ken Cohen

**Affiliations:** United Health Group, Edina, Minnesota, United States; United Health Group, Minnetonka, Minnesota, United States; Optum Labs, Minnetonka, Minnesota, United States; UnitedHealth Group, Minnetonka, Minnesota, United States; United Health Group, Eden Prarie, Minnesota, United States; UnitedHealth Group, Minnetonka, Minnesota, United States; United Health Group, Minnetonka, Minnesota, United States

## Abstract

Alzheimer’s Disease and Related Dementias (ADRDs) are debilitating conditions that impact cognition and function of nearly 5 million adults in the U.S. This study describes the landscape of prescription drugs to treat cognitive and behavioral symptoms of ADRD among Medicare Advantage Part D enrollees with ADRD (Dementia, Alzheimer’s Disease, Parkinson’s, Frontotemporal Dementia, Lewy Body, Vascular Dementia, or Mild Cognitive Impairment). We assessed prevalence of prescription fills in the years pre- and post-ADRD diagnosis. Prescription fills to manage cognitive symptoms, were assessed by acetylcholinesterase inhibitors and NMDA receptor antagonists. Prescription fills to manage behavioral and psychological symptoms of dementia were assessed by antipsychotics, mood stabilizers and anticonvulsants, antidepressants, sedative-hypnotic z-drugs, and benzodiazepines. The final sample (N=161,368) consisted of 79.2% white, 62.2% female with median age 83 (IQR 77-89). Most drug fills increased from pre-diagnosis year to post diagnosis year including Acetylcholinesterase inhibitor (15.3% to 35.5%), NMDA Receptor Antagonists (5.8% to 16.6%), mood stabilizers and anticonvulsants (15.8% to 18.2%), and antidepressants (19.4% to 24.2%). Benzodiazepine use remained stable (13.0% to 13.6%) as did Sedative hypnotic z- drugs (1.9% 1.8%). Results reveal polypharmacy (median of 11 prescriptions post-ADRD diagnosis) and high healthcare utilization (median of 10 specialty types visited post-ADRD diagnosis). This study shows discrepancies between real-world pharmacologic ADRD management and evidence-based practices. This work highlights the need for innovative care models that simultaneously address polypharmacy and optimally coordinated care for people with ADRD.

